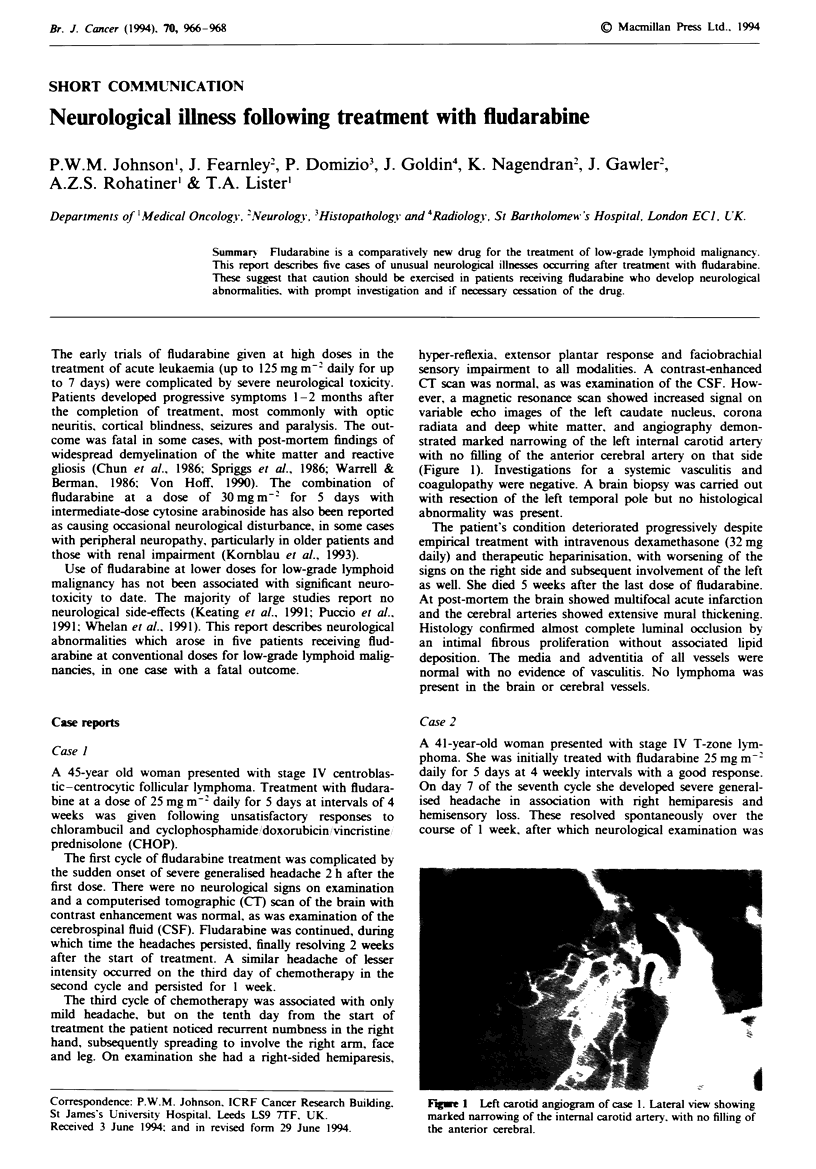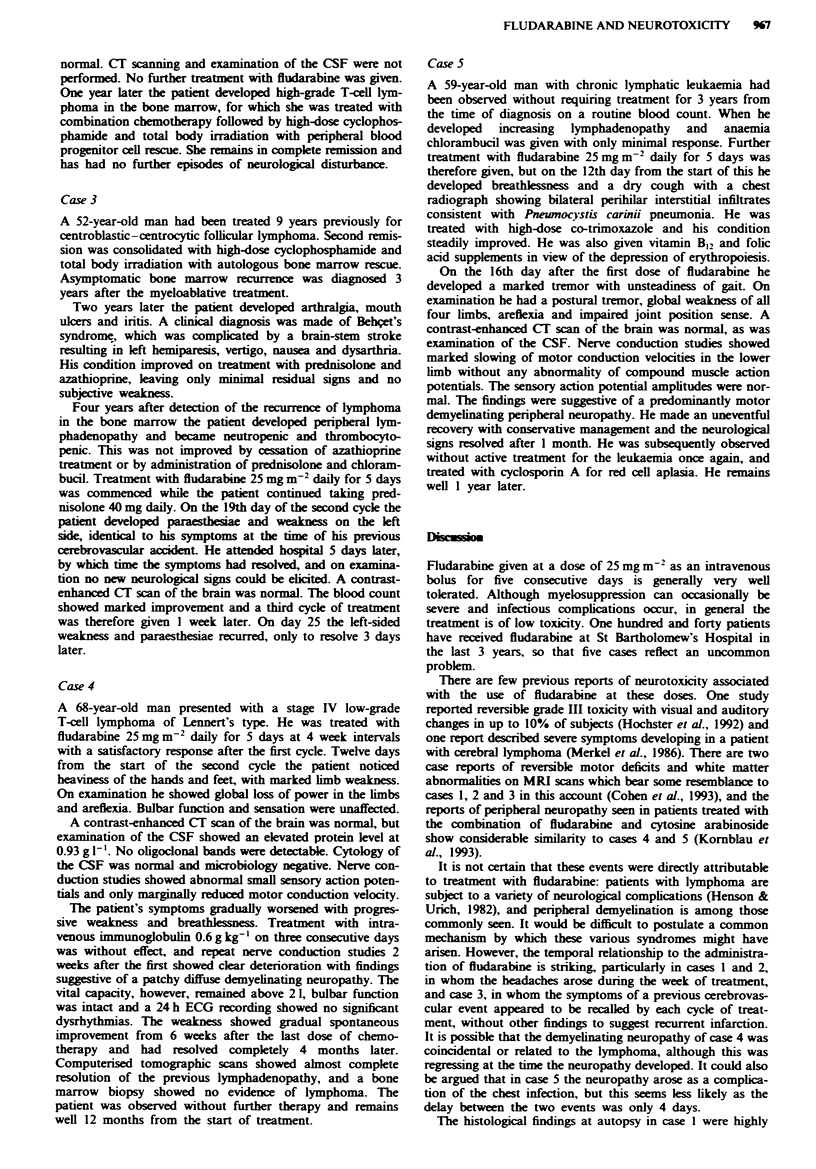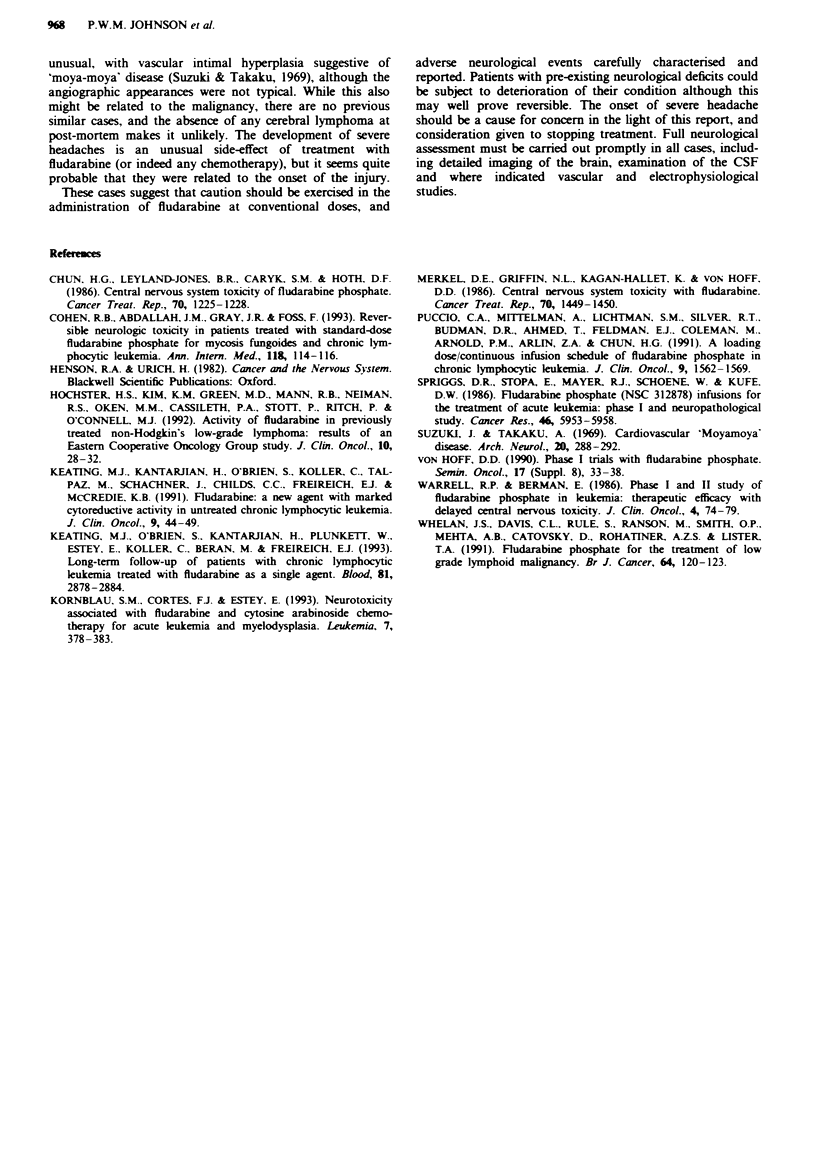# Neurological illness following treatment with fludarabine.

**DOI:** 10.1038/bjc.1994.430

**Published:** 1994-11

**Authors:** P. W. Johnson, J. Fearnley, P. Domizio, J. Goldin, K. Nagendran, J. Gawler, A. Z. Rohatiner, T. A. Lister

**Affiliations:** Department of Medical Oncology, St Bartholomew's Hospital, London, UK.

## Abstract

**Images:**


Br. J. Cancer (1994), 70, 966-968
© Macmillan Press Ltd., 1994

SHORT COMMUNICATION

### Neurological illness following treatment with fludarabine

P.W.M. Johnson[1], J. Fearnley[2], P. Domizio[3], J. Goldin[4], K. Nagendran[2], J. Gawler[2], A.Z.S. Rohatiner[1] & T.A. Lister[1]

*Departments of [1]Medical Oncology, [2]Neurology, [3]Histopathology and [4]Radiology, St Bartholomew's Hospital, London EC1, UK.*

**Summary** Fludarabine is a comparatively new drug for the treatment of low-grade lymphoid malignancy. This report describes five cases of unusual neurological illnesses occurring after treatment with fludarabine. These suggest that caution should be exercised in patients receiving fludarabine who develop neurological abnormalities, with prompt investigation and if necessary cessation of the drug.

The early trials of fludarabine given at high doses in the treatment of acute leukaemia (up to 125 mg $m^{-2}$ daily for up to 7 days) were complicated by severe neurological toxicity. Patients developed progressive symptoms 1–2 months after the completion of treatment, most commonly with optic neuritis, cortical blindness, seizures and paralysis. The outcome was fatal in some cases, with post-mortem findings of widespread demyelination of the white matter and reactive gliosis (Chun *et al.*, 1986; Spriggs *et al.*, 1986; Warrell & Berman, 1986; Von Hoff, 1990). The combination of fludarabine at a dose of 30 mg $m^{-2}$ for 5 days with intermediate-dose cytosine arabinoside has also been reported as causing occasional neurological disturbance, in some cases with peripheral neuropathy, particularly in older patients and those with renal impairment (Kornblau *et al.*, 1993).

Use of fludarabine at lower doses for low-grade lymphoid malignancy has not been associated with significant neurotoxicity to date. The majority of large studies report no neurological side-effects (Keating *et al.*, 1991; Puccio *et al.*, 1991; Whelan *et al.*, 1991). This report describes neurological abnormalities which arose in five patients receiving fludarabine at conventional doses for low-grade lymphoid malignancies, in one case with a fatal outcome.

### Case reports

### *Case 1*

A 45-year old woman presented with stage IV centroblastic–centrocytic follicular lymphoma. Treatment with fludarabine at a dose of 25 mg $m^{-2}$ daily for 5 days at intervals of 4 weeks was given following unsatisfactory responses to chlorambucil and cyclophosphamide/doxorubicin/vincristine/prednisolone (CHOP).

The first cycle of fludarabine treatment was complicated by the sudden onset of severe generalised headache 2 h after the first dose. There were no neurological signs on examination and a computerised tomographic (CT) scan of the brain with contrast enhancement was normal, as was examination of the cerebrospinal fluid (CSF). Fludarabine was continued, during which time the headaches persisted, finally resolving 2 weeks after the start of treatment. A similar headache of lesser intensity occurred on the third day of chemotherapy in the second cycle and persisted for 1 week.

The third cycle of chemotherapy was associated with only mild headache, but on the tenth day from the start of treatment the patient noticed recurrent numbness in the right hand, subsequently spreading to involve the right arm, face and leg. On examination she had a right-sided hemiparesis, hyper-reflexia, extensor plantar response and faciobrachial sensory impairment to all modalities. A contrast-enhanced CT scan was normal, as was examination of the CSF. However, a magnetic resonance scan showed increased signal on variable echo images of the left caudate nucleus, corona radiata and deep white matter, and angiography demonstrated marked narrowing of the left internal carotid artery with no filling of the anterior cerebral artery on that side (Figure 1). Investigations for a systemic vasculitis and coagulopathy were negative. A brain biopsy was carried out with resection of the left temporal pole but no histological abnormality was present.

The patient's condition deteriorated progressively despite empirical treatment with intravenous dexamethasone (32 mg daily) and therapeutic heparinisation, with worsening of the signs on the right side and subsequent involvement of the left as well. She died 5 weeks after the last dose of fludarabine. At post-mortem the brain showed multifocal acute infarction and the cerebral arteries showed extensive mural thickening. Histology confirmed almost complete luminal occlusion by an intimal fibrous proliferation without associated lipid deposition. The media and adventitia of all vessels were normal with no evidence of vasculitis. No lymphoma was present in the brain or cerebral vessels.

### *Case 2*

A 41-year-old woman presented with stage IV T-zone lymphoma. She was initially treated with fludarabine 25 mg $m^{-2}$ daily for 5 days at 4 weekly intervals with a good response. On day 7 of the seventh cycle she developed severe generalised headache in association with right hemiparesis and hemisensory loss. These resolved spontaneously over the course of 1 week, after which neurological examination was

**Figure 1** Left carotid angiogram of case 1. Lateral view showing marked narrowing of the internal carotid artery, with no filling of the anterior cerebral.

Correspondence: P.W.M. Johnson, ICRF Cancer Research Building, St James's University Hospital, Leeds LS9 7TF, UK.
Received 3 June 1994; and in revised form 29 June 1994.

normal. CT scanning and examination of the CSF were not performed. No further treatment with fludarabine was given. One year later the patient developed high-grade T-cell lymphoma in the bone marrow, for which she was treated with combination chemotherapy followed by high-dose cyclophosphamide and total body irradiation with peripheral blood progenitor cell rescue. She remains in complete remission and has had no further episodes of neurological disturbance.

*Case 3*

A 52-year-old man had been treated 9 years previously for centroblastic–centrocytic follicular lymphoma. Second remission was consolidated with high-dose cyclophosphamide and total body irradiation with autologous bone marrow rescue. Asymptomatic bone marrow recurrence was diagnosed 3 years after the myeloablative treatment.

Two years later the patient developed arthralgia, mouth ulcers and iritis. A clinical diagnosis was made of Behçet's syndrome, which was complicated by a brain-stem stroke resulting in left hemiparesis, vertigo, nausea and dysarthria. His condition improved on treatment with prednisolone and azathioprine, leaving only minimal residual signs and no subjective weakness.

Four years after detection of the recurrence of lymphoma in the bone marrow the patient developed peripheral lymphadenopathy and became neutropenic and thrombocytopenic. This was not improved by cessation of azathioprine treatment or by administration of prednisolone and chlorambucil. Treatment with fludarabine 25 mg $m^{-2}$ daily for 5 days was commenced while the patient continued taking prednisolone 40 mg daily. On the 19th day of the second cycle the patient developed paraesthesiae and weakness on the left side, identical to his symptoms at the time of his previous cerebrovascular accident. He attended hospital 5 days later, by which time the symptoms had resolved, and on examination no new neurological signs could be elicited. A contrast-enhanced CT scan of the brain was normal. The blood count showed marked improvement and a third cycle of treatment was therefore given 1 week later. On day 25 the left-sided weakness and paraesthesiae recurred, only to resolve 3 days later.

*Case 4*

A 68-year-old man presented with a stage IV low-grade T-cell lymphoma of Lennert's type. He was treated with fludarabine 25 mg $m^{-2}$ daily for 5 days at 4 week intervals with a satisfactory response after the first cycle. Twelve days from the start of the second cycle the patient noticed heaviness of the hands and feet, with marked limb weakness. On examination he showed global loss of power in the limbs and areflexia. Bulbar function and sensation were unaffected.

A contrast-enhanced CT scan of the brain was normal, but examination of the CSF showed an elevated protein level at 0.93 g $l^{-1}$. No oligoclonal bands were detectable. Cytology of the CSF was normal and microbiology negative. Nerve conduction studies showed abnormal small sensory action potentials and only marginally reduced motor conduction velocity.

The patient's symptoms gradually worsened with progressive weakness and breathlessness. Treatment with intravenous immunoglobulin 0.6 g $kg^{-1}$ on three consecutive days was without effect, and repeat nerve conduction studies 2 weeks after the first showed clear deterioration with findings suggestive of a patchy diffuse demyelinating neuropathy. The vital capacity, however, remained above 2 l, bulbar function was intact and a 24 h ECG recording showed no significant dysrhythmias. The weakness showed gradual spontaneous improvement from 6 weeks after the last dose of chemotherapy and had resolved completely 4 months later. Computerised tomographic scans showed almost complete resolution of the previous lymphadenopathy, and a bone marrow biopsy showed no evidence of lymphoma. The patient was observed without further therapy and remains well 12 months from the start of treatment.

*Case 5*

A 59-year-old man with chronic lymphatic leukaemia had been observed without requiring treatment for 3 years from the time of diagnosis on a routine blood count. When he developed increasing lymphadenopathy and anaemia chlorambucil was given with only minimal response. Further treatment with fludarabine 25 mg $m^{-2}$ daily for 5 days was therefore given, but on the 12th day from the start of this he developed breathlessness and a dry cough with a chest radiograph showing bilateral perihilar interstitial infiltrates consistent with *Pneumocystis carinii* pneumonia. He was treated with high-dose co-trimoxazole and his condition steadily improved. He was also given vitamin $B_{12}$ and folic acid supplements in view of the depression of erythropoiesis.

On the 16th day after the first dose of fludarabine he developed a marked tremor with unsteadiness of gait. On examination he had a postural tremor, global weakness of all four limbs, areflexia and impaired joint position sense. A contrast-enhanced CT scan of the brain was normal, as was examination of the CSF. Nerve conduction studies showed marked slowing of motor conduction velocities in the lower limb without any abnormality of compound muscle action potentials. The sensory action potential amplitudes were normal. The findings were suggestive of a predominantly motor demyelinating peripheral neuropathy. He made an uneventful recovery with conservative management and the neurological signs resolved after 1 month. He was subsequently observed without active treatment for the leukaemia once again, and treated with cyclosporin A for red cell aplasia. He remains well 1 year later.

### Discussion

Fludarabine given at a dose of 25 mg $m^{-2}$ as an intravenous bolus for five consecutive days is generally very well tolerated. Although myelosuppression can occasionally be severe and infectious complications occur, in general the treatment is of low toxicity. One hundred and forty patients have received fludarabine at St Bartholomew's Hospital in the last 3 years, so that five cases reflect an uncommon problem.

There are few previous reports of neurotoxicity associated with the use of fludarabine at these doses. One study reported reversible grade III toxicity with visual and auditory changes in up to 10% of subjects (Hochster *et al.*, 1992) and one report described severe symptoms developing in a patient with cerebral lymphoma (Merkel *et al.*, 1986). There are two case reports of reversible motor deficits and white matter abnormalities on MRI scans which bear some resemblance to cases 1, 2 and 3 in this account (Cohen *et al.*, 1993), and the reports of peripheral neuropathy seen in patients treated with the combination of fludarabine and cytosine arabinoside show considerable similarity to cases 4 and 5 (Kornblau *et al.*, 1993).

It is not certain that these events were directly attributable to treatment with fludarabine: patients with lymphoma are subject to a variety of neurological complications (Henson & Urich, 1982), and peripheral demyelination is among those commonly seen. It would be difficult to postulate a common mechanism by which these various syndromes might have arisen. However, the temporal relationship to the administration of fludarabine is striking, particularly in cases 1 and 2, in whom the headaches arose during the week of treatment, and case 3, in whom the symptoms of a previous cerebrovascular event appeared to be recalled by each cycle of treatment, without other findings to suggest recurrent infarction. It is possible that the demyelinating neuropathy of case 4 was coincidental or related to the lymphoma, although this was regressing at the time the neuropathy developed. It could also be argued that in case 5 the neuropathy arose as a complication of the chest infection, but this seems less likely as the delay between the two events was only 4 days.

The histological findings at autopsy in case 1 were highly unusual, with vascular intimal hyperplasia suggestive of 'moya-moya' disease (Suzuki & Takaku, 1969), although the angiographic appearances were not typical. While this also might be related to the malignancy, there are no previous similar cases, and the absence of any cerebral lymphoma at post-mortem makes it unlikely. The development of severe headaches is an unusual side-effect of treatment with fludarabine (or indeed any chemotherapy), but it seems quite probable that they were related to the onset of the injury.

These cases suggest that caution should be exercised in the administration of fludarabine at conventional doses, and adverse neurological events carefully characterised and reported. Patients with pre-existing neurological deficits could be subject to deterioration of their condition although this may well prove reversible. The onset of severe headache should be a cause for concern in the light of this report, and consideration given to stopping treatment. Full neurological assessment must be carried out promptly in all cases, including detailed imaging of the brain, examination of the CSF and where indicated vascular and electrophysiological studies.

### References

CHUN, H.G., LEYLAND-JONES, B.R., CARYK, S.M. & HOTH, D.F. (1986). Central nervous system toxicity of fludarabine phosphate. *Cancer Treat. Rep.*, **70,** 1225-1228.

COHEN, R.B., ABDALLAH, J.M., GRAY, J.R. & FOSS, F. (1993). Reversible neurologic toxicity in patients treated with standard-dose fludarabine phosphate for mycosis fungoides and chronic lymphocytic leukemia. *Ann. Intern. Med.*, **118,** 114-116.

HENSON, R.A. & URICH, H. (1982). *Cancer and the Nervous System.* Blackwell Scientific Publications: Oxford.

HOCHSTER, H.S., KIM, K.M, GREEN, M.D., MANN, R.B., NEIMAN, R.S., OKEN, M.M., CASSILETH, P.A., STOTT, P., RITCH, P. & O'CONNELL, M.J. (1992). Activity of fludarabine in previously treated non-Hodgkin's low-grade lymphoma: results of an Eastern Cooperative Oncology Group study. *J. Clin. Oncol.*, **10,** 28-32.

KEATING, M.J., KANTARJIAN, H., O'BRIEN, S., KOLLER, C., TALPAZ, M., SCHACHNER, J., CHILDS, C.C., FREIREICH, E.J. & MCCREDIE, K.B. (1991). Fludarabine: a new agent with marked cytoreductive activity in untreated chronic lymphocytic leukemia. *J. Clin. Oncol.*, **9,** 44-49.

KEATING, M.J., O'BRIEN, S., KANTARJIAN, H., PLUNKETT, W., ESTEY, E., KOLLER, C., BERAN, M. & FREIREICH, E.J. (1993). Long-term follow-up of patients with chronic lymphocytic leukemia treated with fludarabine as a single agent. *Blood*, **81,** 2878-2884.

KORNBLAU, S.M., CORTES, F.J. & ESTEY, E. (1993). Neurotoxicity associated with fludarabine and cytosine arabinoside chemotherapy for acute leukemia and myelodysplasia. *Leukemia*, **7,** 378-383.

MERKEL, D.E., GRIFFIN, N.L., KAGAN-HALLET, K. & VON HOFF, D.D. (1986). Central nervous system toxicity with fludarabine. *Cancer Treat. Rep.*, **70,** 1449-1450.

PUCCIO, C.A., MITTELMAN, A., LICHTMAN, S.M., SILVER, R.T., BUDMAN, D.R., AHMED, T., FELDMAN, E.J., COLEMAN, M., ARNOLD, P.M., ARLIN, Z.A. & CHUN, H.G. (1991). A loading dose/continuous infusion schedule of fludarabine phosphate in chronic lymphocytic leukemia. *J. Clin. Oncol.*, **9,** 1562-1569.

SPRIGGS, D.R., STOPA, E., MAYER, R.J., SCHOENE, W. & KUFE, D.W. (1986). Fludarabine phosphate (NSC 312878) infusions for the treatment of acute leukemia: phase I and neuropathological study. *Cancer Res.*, **46,** 5953-5958.

SUZUKI, J. & TAKAKU, A. (1969). Cardiovascular 'Moyamoya' disease. *Arch. Neurol.*, **20,** 288-292.

VON HOFF, D.D. (1990). Phase I trials with fludarabine phosphate. *Semin. Oncol.*, **17** (Suppl. 8), 33-38.

WARRELL, R.P. & BERMAN, E. (1986). Phase I and II study of fludarabine phosphate in leukemia: therapeutic efficacy with delayed central nervous toxicity. *J. Clin. Oncol.*, **4,** 74-79.

WHELAN, J.S., DAVIS, C.L., RULE, S., RANSON, M., SMITH, O.P., MEHTA, A.B., CATOVSKY, D., ROHATINER, A.Z.S. & LISTER, T.A. (1991). Fludarabine phosphate for the treatment of low grade lymphoid malignancy. *Br J. Cancer*, **64,** 120-123.